# Preparation, Characterization and Evaluation of Sun Protective and Moisturizing Effects of Nanoliposomes Containing Safranal

**Published:** 2011

**Authors:** Shiva Golmohammadzadeh, Fatemeh Imani, Hossein Hosseinzadeh, Mahmoud Reza Jaafari

**Affiliations:** 1 *Nanotechnology Research Centre, Mashhad, University of Medical Sciences, Mashhad, Iran*; 2 *School of Pharmacy, Mashhad University of Medical Sciences, Mashhad, Iran*; 3 *Pharmaceutical Research Centre, Mashhad University of Medical Sciences, Mashhad, Iran*; 4 *Biotechnology Research Centre, Mashhad University of Medical Sciences, Mashhad, Iran*

**Keywords:** Liposomes, Moisture, Saffron, Safranal, SunProtection Factor

## Abstract

**Objective(s):**

The objective of this study was to prepare, characterize and evaluate the nanoliposomes containing safranal as a natural sunscreen and moisturizer factor.

**Materials and Methods:**

The experimental formulations included homosalate reference, nanoliposomes containing 0.25, 0.5, 1, 2, 4 and 8% safranal and empty liposomes. The liposomes were prepared using fusion method and homogenization. Homosalate reference was prepared according to FDA standard. Sun protection factors (SPF) of the formulations were determined by two *in vitro *methods; diluted solution transmittance method and transpore tape method. Studies of *in vitro* penetration of the formulations across mouse skin were carried out with diffusion cells. The percentage of safranal penetrated and retained in the skin was determined for the formulations up to 24 hr. The amount of the moisture contents of the skin before application and after 30-minute, 1, 3 and 5 hr post-application of the formulations were measured in human volunteers using Corneometer.

**Results:**

The results indicated that, the SPF of liposomes containing 8% safranal (Lip-Safranal 8%) was significantly higher than 8% homosalate reference. The proportion of Lip-Safranal 1% that penetrated the skin was low. There was no significant difference between the skin moisture contents after application of Lip-Safranal 1 and 4% and empty liposomes during the 7 hr post-application period.

**Conclusion:**

These results showed that in equal concentrations, Lip-Safranal could act as a better antisolar agent compared to homosalate and have no moisturizing effect in 1 and 4% concentrations.

## Introduction

The harmful effects of solar radiation are caused by ultraviolet radiation (UVR) part of the solar rays. UVA and UVB are mainly responsible for skin pathologies such as sunburns, cutaneous degeneration, photosensitivity, phototoxicity, photo-aging, immunosuppression and skin cancer (1-3).

The growing awareness of the damage that UVR might cause on human health has led to increasing use of sunscreen products (1,2). The efficacy of a sunscreen is usually expressed by the Sun Protection Factor (SPF). Higher SPF values result in more effective products in preventing sunburn. Sunscreen products are usually applied superficially to large skin areas; therefore, penetration of the sunscreen’s ingredients may occur, which is not desirable (4-6). Irritation may also occur with some chemical sunscreens (7).

Nowadays, using the natural products that can absorb UVR is of great interest in sunscreen products. This is because of the benefits of these products, more acceptability by the users; also the low probability of the systemic absorption. (8). Natural substances extracted from plants have recently been considered as potential sunscreen resources because of their UV absorption and their antioxidant activity (8-13). 

Saffron is the dried stigmas of a flower scientifically identified as *Crocus sativus. *It is a perennial stemless herb widely cultivated in Iran and some other countries such as India, Spain and Greece (14). Pharmacological studies have revealed that saffron extract has antitumor, radical scavenging properties (15-17), as well as antinociceptive, antiinflammatory (18), anticonvulsant (19), and antidepressant effects (20,21). The main aroma factor in saffron is safranal, which comprises about 60% of the volatile components of the saffron (17). The investigations demonstrate that saffron and its active constituents like safranal have anti-tumor, antioxidant and antigenotoxic effects (15-18).

Organic sunscreens are generally aromatic compounds conjugated with a carbonyl group (26). In our previous study, because of the advantages of saffron besides having many aromatic and flavonoid compounds such as kaempherol and quercetin, the SPFs of the lotions containing ground saffron were evaluated and established (27). In this study, according to the aromatic conjugated with a carbonyl group structure of safranal and its antioxidant activity besides its UV absorption spectrum, the possibility of using this component as a sunscreen, was investigated. 

Many factors are involved in the delivery of the drugs and cosmetics into the skin from topically applied formulations. Liposomes are preferable in sunscreen formulations. They exhibit unique features by offering easy delivery, no interference with vision, stabilizing the drug, excellent reservoirs for drug loading and water resistance properties (28-30). Several factors; such as, physicochemical properties of the drug and other ingredients, lamellarity, lipid composition, charge, size, vehicle, mode of application and total lipid concentrations have been proven to influence drug deposition into the skin layers. The other advantage of a liposome-based drug product is that fewer drugs need to be administered. Thus, the probability of systemic absorption and adverse drug reactions is reduced (28-32).

Another appropriate effect of a sunscreen product is producing a moisturizing effect. Corneometers have gained worldwide acceptance as an efficient instrument to measure the water content in the stratum corneum (SC) (33). 

The aim of this research was to characterize the liposomes containing safranl (Lip-Safranal) and determine and compare the SPF values of these liposomal formulations by two *in vitro *methods and evaluate the moisturizing effects of the formulations on the skin of human volunteers using Corneometer.

## Materials and Methods

***Reagents and chemicals***

Homosalate, cholesterol and vitamin E, were purchased from Merck (). Lanolin, white petrolatum, stearic acid, propylparaben (PP), methylparaben (MP), disodium EDTA, propylene glycol, triethanolamine, N-[2-hydroxyethyl] piperazine-N_-[2-ethanesulfonic acid] (HEPES) and safranal were purchased from Sigma (USA). Soya phosphatidylcholine (Soya PC) was obtained from the Avanti Polar Lipids (). All solvents used in this study were high performance liquid chromatography (HPLC) grade. All chemicals were of the purest grade available. 

***Preparation of the homosalate reference as the standard sunscreen***

A standard sunscreen formulation was needed for ensuring reproducible results in SPF determinations. This standard was prepared according to the FDA and Australian standards. According to FDA, the SPF of this standard preparation is 4.47±1.279 (21,27). Homosalate (8%), lanolin (5%), white petrolatum (2.5%), stearic acid (4%) and propylparaben (0.05%) were melted at 77 - 82°C as the oil phase. Methylparaben (0.1%), disodium EDTA (0.05%), propylene glycol (5%), triethanolamine (1%) and water up to 100% were heated as the aqueous phase with constant stirring. The aqueous phase was added to oil phase, and the mixture was stirred until it cooled down to room temperature (34-36).

***Preparation of liposomes containing safranal (Lip-Safranal)***

Lip-Safranal (0.25, 0.5, 1, 2, 4 and 8%) was prepared by fusion method (28). The lipid components consisted of Soya PC (15%), cholesterol (2%), vitamin E (0.3%), propylene glycol (7%), MP (0.1%) and PP (0.02%) were melted at about 75°C (melted lipid). When melted lipid cooled down to 50˚C then Oleic acid (1%) and safranal were added and mixed completely. HEPES buffer (10 mM, pH 6.5) and triethanolamine (0.5%) up to 100% were heated separately at 75˚C and was added to the previously heated melted lipid and stirred vigorously until it cooled down to room temperature. The final products were then homogenized with a homogenizer (Ultra-Turrax IKA T10; IKA Werke GmbH & Co. KG, ) for 3 min at 11,500 rpm, 2 min at 14,500 rpm, 1 min at 20,500 rpm and 1 min at 30,000 rpm. The same procedure was used to prepare control empty liposomes, except for omitting the safranal. 

***Characterization of liposomes***

The average particle size and charge of the liposomes were measured in triplicate by the use of dynamic light scattering (ZetaSizer Nano-ZS; Malvern Instruments Ltd., ).. Liposomal preparations were characterized 12 hr after preparation. For particle size measurements, liposomal suspensions were properly diluted with HEPES buffer in order to avoid multiscattering phenomena. For surface charge determination, liposomal dispersions suitably diluted with MOPS buffer were dropped into the Zetamaster electrophoretic cell and the Z potential was determined by electrophoretic mobility measurement (37,38).

Liposomes encapsulation efficiency was determined indirectly, separating the non-entrapped drug from drug-loaded liposomes by dialysis experiments. According to a previously developed method (38), dispersion of 100 mg of drug-loaded liposomes to 1 ml HEPES buffer was prepared and placed into a dialysis bag of cellulose acetate (Spectra/Por®, MW cut-off 12000, Spectrum, Canada) immersed in a closed vessel containing 45 ml of HEPES buffer at 20^ ◦^C, and magnetically stirred at 30 rpm. Samples, withdrawn at time intervals, were replaced with equal volumes of fresh solvent and spectrometrically analyzed (UV 1601 Shimadzu) (37). The maximum absorption of the safranal was obtained as 310 nm. The percentage of encapsulation efficiency (EE %) was calculated according to the following equation:


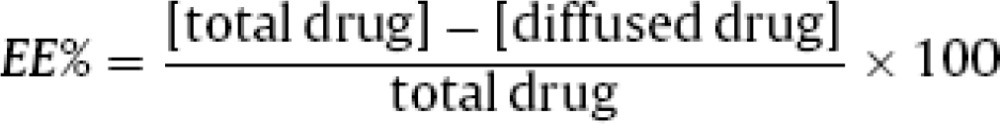


Each result was the mean of at least three separate experiments.

***SPF*** ***determination of the formulations by diluted solution transmittance method***

All samples (1 g) were weighed, transferred to a 100 ml volumetric flask, diluted to volume with ethanol, mixed for 5 min, and then filtered through Whatman filters. A 5 ml sample was transferred to a 25 ml volumetric flask and diluted to volume with ethanol. The absorption values were obtained in the range of 290 to 320 nm (every 5 nm) and three determinations were made at each point. Then, Mansur equation was used to determine the SPF values of the formulations. The introduced equation is as follows: 


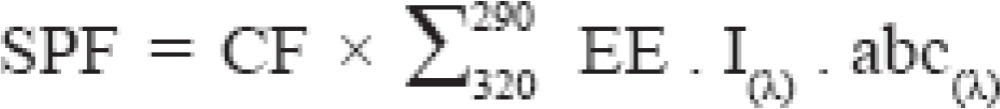


In this equation, CF= 10 (correction factor), EE (λ) = erythemogenic effect of radiation at wavelength λ, I(λ) = intensity of solar light at wavelength λ, and abs(λ)=absorbance of sample at wavelength λ. The values for the term “EE × I” are constants, which were determined by Sayre *et al* and are shown in Table 1(39,40). 

***SPF determination of the formulations by transpore tape method***

The principle of this method is to measure the spectral transmittance of UVR through a sample of a surgical tape which is called transpore tape with and without the sunscreen applied. This substrate was introduced first by Diffey and Robson (41,42). A piece of transpore tape was placed over the quartz cell and then 2 mg/cm^2^ of sunscreen was applied by spotting the sunscreen at several sites over the entire application area. A gloved finger was used to achieve as uniform thickness as possible with a circular light rubbing motion. After 15 min the transmission was measured by UV spectrophotometer. The data of transmittances were set to five nm intervals from 290 nm to 400 nm. The SPF was predicted from the following equation; 


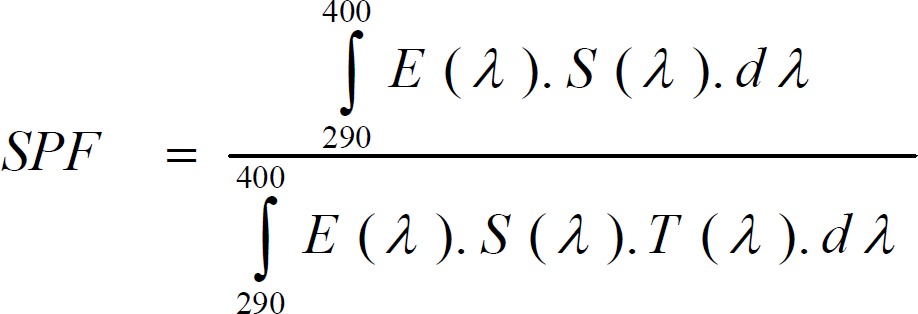


In this equation: E (λ): Relative erythemal spectral effectiveness**; **S (λ): Solar spectral irradiance (Wm^-2^nm^-1^); T (λ): Spectral transmittance of the sample (as measured on the UV-1000S)

The concentrations of the safranal liposomes were selected to obtain minimal sunburn protection (Table 2).

***Moisture content measurement of the skin***

The moisture content of the skin was measured by Corneometer (Courage & Khazaka, ). In practice, the technique is used to measure the difference in the hydration state of SC before and after application of cosmetic or other skin treatments. The test was carried out on six volunteers with normal skin, aged between 20 and 35 at room temperature. Before the measurements, they were given time to adapt to the room conditions without covering the measuring sites with clothes. On the day of examination, the skin was not washed, and nothing was applied to the skin surface. The volunteers were instructed not to apply any preparation to the site to be examined one week before the investigation. In all subjects, the tested sites of the skin were free of eczematous involvement. All measured values were expressed as the median of three recordings. The measurements were carried out on exactly the same sites. The testing site of the skin was the middle of the forearm (33, 43-46). The moisture content of the skin was measured without any application of the products and after 0.5, 1, 3 and 5 hr after application of the Lip–Safranal 1 and 4%.

***Cell diffusion study***

Jacketed Franz cells with a receiver volume of 25 ml were used, and every experiment was conducted in triplicate at 37 °C. HEPES buffer of pH 6.5 was used as the receiver medium. A suitable size of full-thickness skin of a BALB/c mouse was cut and mounted in the Franz cell, with the SC side facing upward. The mouse was properly shaved with electric clippers on the day before the experiment. The membranes were initially left in the Franz cells for 30 min in order to facilitate hydration. Subsequently, 1 g of the liposomal formulation was deposited onto each membrane surface. A 5 ml aliquot was withdrawn from each receiver solution at 1, 2, 4, 8 and 24 hr intervals and replaced with the same volume of HEPES buffer. Aliquots of the collected samples were analyzed for their safranal by the spectrophotometric method. The derived concentration values were corrected by using the equation:

$\mathrm{Mt}\left(n\right)=\mathrm{Vr}\times \mathrm{Cn}+\mathrm{Vs}\times \sum \mathrm{Cm}$

where *Mt *(*n*) is the current cumulative mass of drug transported across the skin at the time *t*, *n *is the number (times) of sampling, *Cn *is the current concentration in the receiver medium,  *Cm *is the summed total of the previously measured concentrations, *Vr *is the volume of the receiver medium, and *Vs *corresponds to the volume of the sample removed for analysis. For the determination of the amount of liposome retained in the skin, at the end of the experiment, the amount of the formulation remaining on the surface of the membrane was collected and assayed for safranal (47-49).

***Statistical analysis***

One-way ANOVA was used to assess the significance of the differences between groups. In case of significant F value multiple comparison Tukey-Kramer tests were used to compare the means of different treatment groups. Results with *P*< 0.05 were considered to be statistically significant. 

## Results

***Characterization of the liposomes***

In this study, six concentrations of Lip-Safranal were prepared. Mean diameters of Liposomes determined by PSA has been shown in Table 3. The differences between the sizes were not significant (*P*> 0.05) but the zeta potential of 0.25%, was significantly higher than 2% and 4% (*P*< 0.01) and 8% (*P*< 0.001). Liposomes exhibited a slight increase in their size after 1, 2 and three- month storage at 4 ºC but this was not statistically significant (*P*> 0.05). 

Liposomes with low encapsulation efficiency were achieved. The encapsulation efficiency for Lip-Safranal 1% was 1.46±0.2% and the percentage releases of 71.03±5.11%, 80.67±9.55% and 84.06±11.42% were obtained after 12, 24 and 36 hr respectively (Table 4).

Statistical analysis showed that encapsulation efficiency was independent of liposome size and the differences between groups were not significant (*P*> 0.05).

***Cell diffusion study***

Studies of the *in vitro* penetration of the formulations across mouse skin were carried out with diffusion cells. The percentage of 

**Table 1.** The values of EE_(λ)_. I_(λ)_ for conclusion of the SPF values in transmittance method

λ (nm)	EE (λ). I(λ)
290	0.0150
295	0.0817
300	0.2874
305	0.3278
310	0.1864
315	0.0839
320	0.0180

**Table 2.** Sunscreen potency assessment by the FDA, based on sun protection factor (SPF)

Sunburn protection	SPF
Minimal	2-12
Moderate	12-30
High	≥ 30

**Table 3.** Mean diameter and zeta potential of different Lip-Safranal concentrations (± SD, n= 3)

Safranal concentrations in liposomes (w/v)	Empty liposomes	0.25%	0.5%	1%	2%	4%	8%
Mean diameter (nm)	102.3±2.63	104.5±7.69	110.1±2.20	118.57±20.93	128.6±49.40	135.9±21.45	90.2±31.80
Zeta potential (mV)	-49.3±3.20	-52.3±3.60	-47.3±1.50	-46.5±3.50	-37.4±4.40	-38.4±5.10	-34.8±2.30
Polydispersity Index	0.215± 0.11	0.304±0.12	0.255±0.06	0.336±0.03	0.109±0.01	0.134±0.05	0.253±0.07

**Table 4.** The percentage release of Lip-Safranal concentrations (±SD, n= 3)

Time	12 hr	24 hr	36 hr
Amount of safranal (µg)	355.13±25.54	48.21±22.23	16.98±9.34
Accumulation amount of safranal (µg)	355.13±25.54	403.34±47.77	420.32±57.11
Percentage release of safranal	71.03±5.11	80.67±9.55	84.06±11.42

safranal penetrated and retained in the skin was determined for the formulations for up to 24 hr. The proportion of Lip-Safranal 1% that penetrated the skin was 8.06±0.48 % and the proportion of safranal in the liposomes that was retained on the skin was 0.47± 0.42 %.

***SPF*** ***determination of the formulations by diluted solution transmittance method and transpore tape***

**Figure 1.**
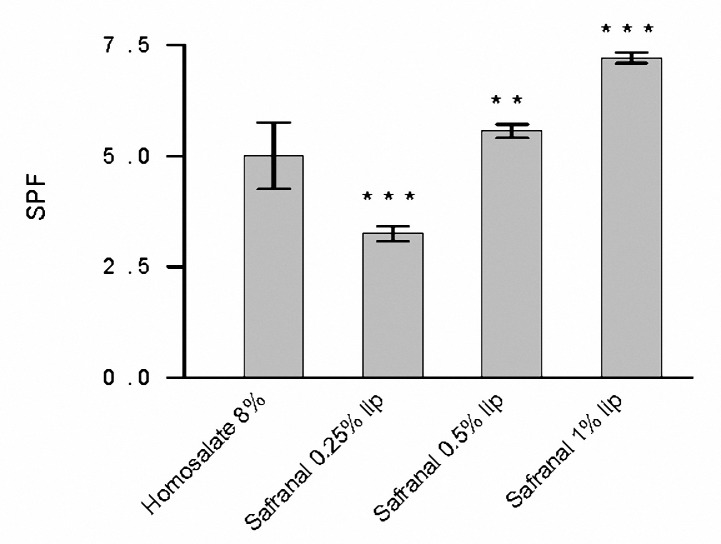
The SPF values of Lip-Safranal (0.25, 0.5 and 1%) and homosalate reference determined by diluted solution transmittance method. Values are mean±SD, n= 3; **P< 0.01, ***P< 0.001.

**Figure 2.**
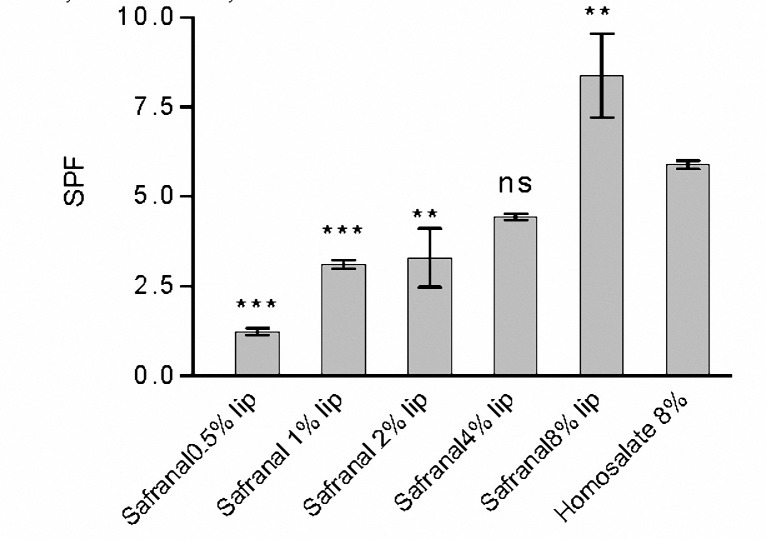
The SPF values of Lip-Safranal (0.5, 1, 2, 4 and 8%) and homosalate reference determined by transpore tape method. Values are the mean±SD, n= 3; **P< 0.01, ***P< 0.001.

Six concentrations of Lip-Safranal were evaluated by UV spectrophotometry using Mansur equation and transpore tape methods (39, 41,42). The SPF values of the 8% homosalate reference and the Lip-Safranal 0.25, 0.5, 1, 2, 4 and 8% were measured. Figures 1, 2 show the SPFs of liposomal and homosalate reference formulations by these two *in vitro* methods. There was no significant difference between the values obtained for SPF of homosalate reference by two *in vitro* methods and *in vivo *studies with *P*> 0.05 (6,35).

According to the diluted solution transmittance method, the SPF values of Lip-Safranal 0.5 and 1% were significantly higher than 8% homosalate reference (*P*< 0.05, Figure 1). The SPF of Lip-Safranal 2, 4 and 8% were not accurately obtained as the absorptions was higher than 1. These results show that in very low concentrations, safranal can act as a better antisolar agent compared to homosalate.

There was no significant difference in the SPF values of Lip-safranal 4% and 8% homosalate reference by transpore tape method. However, the SPF of Lip-safranal 8% was significantly higher than 8% homosalate reference (*P*< 0.05). The SPF of the empty liposomes was obtained as 0.98±0.003. 

All of the formulations were stable except for the Lip-Safranal 8% which was returned into two phases after 2 weeks. 

These results show that in equal concentration, safranal can act as a better antisolar agent compared to homosalate. The differences in the results obtained from the two *in vitro* methods will be discussed in the discussion.

***Measurement of the moisture content of the SC following application of Lip-Safranal and empty liposomes***

**Figure 3.**
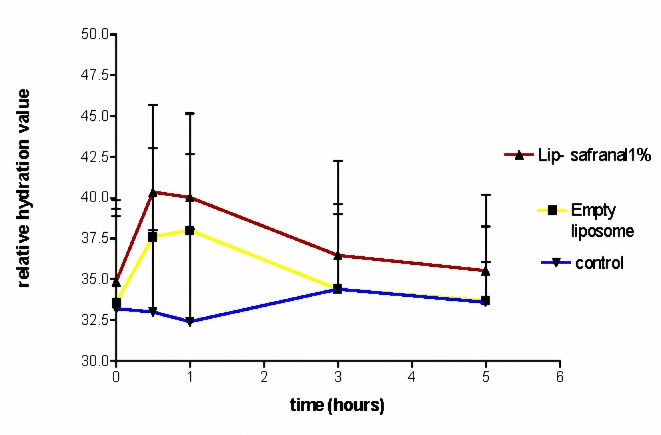
The relative hydration value of Lip-Safranal 1%, Empty liposome and control (Skin hydration without any application).

**Figure 4.**
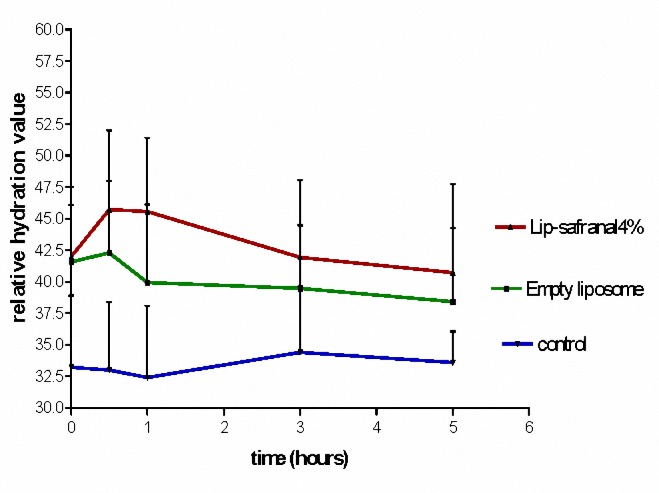
The relative hydration value of Lip-Safranal 4%, Empty liposome and control (Skin hydration without any application).

The water contents of Lip-Safranal 1 and 4% and the empty liposomes were measured using Corneometer CM 825. The Corneometer was calibrated to the baseline value for each subject before application of the formulations on the skin. During the first 30 min after application, the water contents were usually higher than normal. Measurement of the water content of skin at this time (fist 30 min) may result in erroneous data (36, 45,46). Therefore, the first measurement was scheduled at 30 minute after application. The water content of skin was measured 0.5, 1, 3 and 5 hr post-application of Lipo-Safranal 1 and 4%, compared to the baseline which was the value before application of the product. All the tested formulations significantly increased the moisture content of the skin compared to control, in all the tested point times (*P*< 0.01), but there was no significant difference (*P*> 0.05) between the skin moisture contents after application of Lip-Safranal 1 and 4% and the empty liposomes during 5 hr of measurements (Figure 3, 4). Figure 3 shows the relative hydration values for the readings of the test sites measured for Lip-Safranal 4% and empty liposomes at 0.5, 1, 3, and 5 hr post application in relation to the baseline. The trend of the curves in all the treatment groups was nearly the same. At 30 min application, the highest water content was observed; however, there was no significant difference between Lip-Safranal and empty liposomes. After 1 hr, the moisture contents were decreased in all the formulations. After 5 hr of application, the moisture content of the skin for all of the preparation was almost the same, they reached nearly the same degree of hydration and there was no significant difference among them. 

## Discussion

The decrease in intensity of the UVR reaching the skin by sunscreens may reduce the risk of sun-induced skin cancer (50). The efficacy of a sunscreen is usually expressed by the SPF (2, 3, 34-36). Most of the published studies on the determination of SPF have adopted an *in vivo *method based on experiments on human skin, which is very time-consuming and expensive and have human ethical issues. Therefore, developing an *in vitro *method which correlates well with *in vivo *methods is of interest to researchers as an attempt to find a substitute for *in vivo *methods (1, 2,51). Regarding sun-care experiments, it is also a safety issue, since only positive *in vitro *responses will direct the future of the *in vivo *tests (52). Results of several studies indicate that liposomes have been reported as a carrier for active cosmetic ingredients such as humectants (36,53) and sunscreens (30,36).

In this study, the 8% homosalate reference and Lip-Safranal 0.5, 1, 2, 4 and 8 % were prepared. The difference between the sizes was not statistically significant (*P*> 0.05), however the zeta potential of the preparations has been decreased during the increasing of the safranal concentrations. Minus zeta potential of the empty liposomes is due to the oleic acid in bilayer and when safranal is added the oleic acid in the bilayer would be diluted.

As the safranal is placed in the bilayer of the liposomes, diluting the preparations will produce decreasing the zeta potential of the nanoliposomes. The SPF values of the formulations were determined by two *in vitro *methods (39, 41,42). The SPF of Lip-Safranal 0.5 and 1% were significantly higher than 8% homosalate reference (*P*< 0.05) by diluted solution transmittance method. However, the results obtained from transpore tape method indicated that there was no significant difference between the SPF values of Lip-Safranal 4% and that of 8% homosalate reference. These results also indicated that the SPF of Lip-Safranal 8% was significantly higher than 8% homosalate reference (*P*< 0.05). At first, the SPFs of different concentrations of safranal (0.25, 0.5 and 1%) were determined by diluted solution method as safranal dissolves readily in ethanol. These concentrations were selected to obtain minimal sunburn protection (SPF 2-12) according to FDA standard (Table 2). The SPFs of safranal (2, 4 and 8%) were not obtained by this method as the absorptions were higher than 1. As this situation is totally different from that in which a sunscreen agent is applied directly to the skin, it shows a poor correspondence with some sunscreen’s SPFs especially high ones. Despite this, it is still considered to be useful for a preliminary assessment due to its simplicity (1, 2,6). Since safranal is a volatile component, the transpore tape method was also carried out. The results obtained from the second method are more accurate and reliable, as the transpore tape has uneven topography that distributes the sunscreen in a way similar to human skin and imitates the real situation (15 minutes waiting before SPF determination) (14). After 15 min, some of the safranal will evaporate, and the concentrations will decrease in the formulations. Therefore, we required more safranal concentrations to obtain minimal sunburn protection (SPF 2-12) according to FDA standard (Table 2). Thus safranal with concentrations; 0.5, 1, 2, 4 and 8% were prepared. The proposed UV spectrophotometric methods are simple, rapid and use low cost reagents. They can be performed both during the production process and on the final product (39). These results showed that safranal can act as a better antisolar agent compared to homosalate. High SPF value of Lip-Safranal may be related to the aromatic conjugated with a carbonyl group structure of safranal. 

In recent years, natural compounds have gained considerable attention as UV protective agents due to the presumable safe utilization, ecological issues, and minimal side effects besides their antioxidant activity (54,55). Plant extracts, due to presence of a wide range of phenolic acids, flavonoids, and high molecular weight polyphenols, usually cover the full range of UV wavelengths (54, 56,57). Safranal has the aromatic conjugated with a carbonyl group structure and antioxidant activity besides a good UV absorption spectrum. Therefore, the possibility of using the safranal as a sunscreen was investigated in this research. 

In a recent review, Abdullaev and Espinosa- focused on the anticancer activity of saffron and its principal ingredients (17).  

From the results obtained in our previous study, saffron can be used as a natural UV absorbing agent. The 4% saffron lotion showed an SPF value equivalent to the 8% homosalate lotion reference by an *in vitro *method. (27).

Topical application of *Culcitium reflexum *extracts in the form of a gel proved to exert a significant *in vivo *protection against the UV-induced skin erythema in healthy human volunteers. The flavonoid fraction of *Sedum telephium* leaf extracts also appears to possess potent protective effects against UV-induced skin erythema in human volunteers (13,54). 

One approach to protect human skin against the harmful effects of UVR is to use antioxidants as photo-protective. According to our results safranal could act as a better antisolar agent compared to homosalate, besides it has also antitumor and antioxidant activities (16, 17,58)

Ramon *et al. *showed that liposomes could be regarded as alternatives to conventional oil/water emulsions in the formulations of lipidic sun filters. When liposomes with a composition and structural organization similar to that of the SC lipids are used the skin penetration is retarded (30). As the intercellular lipids are important in controlling the percutaneous absorption, liposomes may mix with the intercellular lipids and produce a sustained release carrier system that acts as a reservoir for sunscreen; therefore, the sunscreen remains longer on the outermost layers of the skin (29). This property is essential for sunscreen agents because the amount remained inside the SC maybe directly related to its sun protection value (4,59). In the current study, liposomes were selected as a drug delivery system for safranal because of these benefits and water resistance property (6,28). Liposomes in the proper formulations and sizes have been shown to be able to accumulate in the skin (29, 30, 32,47). 

In our study, as the dialysis is a dynamic system, and the safranal evaporates during dialysis and releases to the buffer, the encapsulation efficiency is expected to be more than 1 %, which was discussed above. Therefore, the liposomes were used without purification.

In this study, soybean PC (SPC) was used for liposome preparations. SPC contains polyunsaturated fatty acids like linoleic acid, which are beneficial for healthy skin. Furthermore, formulations prepared by SPC increase the skin humidity (6, 60). In the liposome formulation cholesterol was included to stabilize the lipid bilayer and decrease the leakage of encapsulated drugs and vesicle aggregation (6,61). Vitamin E was used to prevent SPC oxidation, PP and MP were used as microbial preservatives, and HEPES to control the pH of the liposomal formulations to achieve maximum stability (61).

In this research, fusion method was used to prepare the topical safranal liposomes. The fusion method is one of the more suitable methods for the preparation of these liposomes, as it provides homogeneous liposomes. The fusion method is simple, efficient, and reproducible. It is devoid of organic solvents like chloroform; and yields homogeneous liposomes with high encapsulation efficiencies (62).

In our study, liposomes with low encapsulation efficiency were prepared and no crystallization of either formulation was observed during storage. Furthermore, liposomes prepared by this method showed enough viscosity that they could be applied directly on the skin without mixing the liposomal formulation with other bases.

Some studies showed that sunscreen with smaller sizes can have more sun protective effects (63). In this study, topical Lip-Safranal prepared by the fusion method plus homogenization provided liposomes of submicron sizes (Table 2). Analysis of the particle size distribution showed that the average size of most of the population of Lip-Safranal was less than 150 nm (according to the average size by the number;Table 2). Furthermore, the results of the Franz diffusion cell studies across mouse skin showed low percentage of penetration and retention in the skin when the formulations were used (Figure 1), which cannot prove that these vesicles possess the high penetration ability as the safranal is very volatile.

In this research, the water contents of the skin were measured by Corneometer CM 825 0.5, 1, 3 and 5 hr post-application of the Lip-Safranal 1 and 4% and empty liposomes as well as the control without application (Figures 3, 4). Various methods have been summarized by Fluhr *et al* (64) for measuring the hydration state of the SC (stratum corneum). Common techniques for evaluating moisturizer efficacy are as follows: visual techniques (photography, video microscopy, expert visual grading and subject self-assessment), skin hydration measurement (corneometer), skin barrier function (transepidermal water loss measurement) and skin elasticity studies. Among these tests Corneometer has been used more widely (33). 

In our study, there was no significant difference in skin moisture contents between 5 hr after application of Lip-Safranal 1 and 4% and the empty liposomes. For each formulation, there was a significant increase in moisture content after 30 minute. After 1 hr, the moisture contents were decreased in all of the formulations. All the increases in water content after application of the formulations were significantly more than that of the control. The shapes of the curves in all samples were nearly the same. 

All the tested formulations significantly increased the moisture content of the skin compared to control, but there was no significant difference in skin moisture content between the groups applying the Lip-Safranal or the emty liposomes. This indicates that the increase in water content is due to the liposome and safranal does not have any remarkable moisturizing effect. The liposomes due to their lipophilic structure and their similarity to SC lipids can improve the moisture content. Liposomes provide their own water content and share the water with the skin. The problems of loss of water migrating from the underlying tissues can be resolved by using the liposomes.

Application of humectants to the skin alone is unsatisfactory, since they are not substantive to the skin; they are water soluble and are readily rinsed off (65).

In a patent filled by Unilever Brothers, they used humectants entrapping liposomes in cosmetic creams. These humectants were glycerin, urea and sodium pyroglutamate. Their results showed that the humectants entrapped liposomes absorb great quantities of water. Some moisturizers are designed to promote water retention by their hygroscopic nature while others are designed to prevent water loss from the skin surface by providing an occlusive film or by supplying SC-like lipids (66). The lack of any remarkable moisturizing effect in safranal may be due to the lack of occlusive properties of safranal.

## Conclusion

The results of this study indicated that safranal can be used as a natural UV-absorbing agent. The SPF of Li-Safranal 8% was significantly higher than 8% homosalate reference. There was no statistically significant difference between the skin moisture contents after application of the liposomes containing safranl or empty liposomes. 
